# Supplementary material for: A high-resolution mRNA expression time course of embryonic development in zebrafish
Source: eLife. 2017 Nov 16;6:e30860. doi: 10.7554/eLife.30860 (PMC5690287; doi:10.7554/eLife.30860)
Supplement: Supplementary file 6. [file elife-30860-supp6.zip › biolayout-clusters-files/Cluster229-genes.html]

Cluster229


# Cluster229: Genes

| Ensembl ID | Gene Name | Chr | Start | End | Biotype |
| --- | --- | --- | --- | --- | --- |
| ENSDARG00000016496 | cdk8 | 24 | 21369109 | 21394170 | protein\_coding |
| ENSDARG00000061204 | ddx20 | 8 | 28224074 | 28240640 | protein\_coding |
| ENSDARG00000040650 | gps1 | 3 | 36104458 | 36117301 | protein\_coding |
| ENSDARG00000044282 | smc5 | 10 | 15659969 | 15686821 | protein\_coding |
| ENSDARG00000014770 | usp4 | 6 | 42347469 | 42371650 | protein\_coding |
| ENSDARG00000071487 | zgc:152948 | 21 | 1603862 | 1618590 | protein\_coding |
